# Case report: Prenatal diagnosis of Kagami–Ogata syndrome in a Chinese family

**DOI:** 10.3389/fgene.2022.959666

**Published:** 2022-08-11

**Authors:** Junjie Hu, Ying Zhang, Yanmei Yang, Liya Wang, Yixi Sun, Minyue Dong

**Affiliations:** ^1^ Women’s Hospital, School of Medicine, Zhejiang University, Hangzhou, Zhejiang, China; ^2^ Key Laboratory of Reproductive Genetics (Zhejiang University), Ministry of Education, Hangzhou, Zhejiang, China; ^3^ Key Laboratory of Women’s Reproductive Health of Zhejiang Province, Women’s Hospital, School of Medicine, Zhejiang University, Hangzhou, Zhejiang, China

**Keywords:** Kagami–Ogata syndrome, Temple syndrome, differentially methylated region, imprinting disorder, prenatal diagnosis

## Abstract

The aim of this work was to explore the genetic cause of the proband (Ⅲ2) presenting with polyhydramnios and gastroschisis. Copy number variation sequencing (CNV-seq), methylation-specific multiplex PCR (MS-PCR), and methylation-specific multiplex ligation-dependent probe amplification (MS-MLPA) were used to characterize the genetic etiology. CNV-seq revealed a deletion of 732.26 kb at 14q32.2q32.31 in the proband (Ⅲ2) and its mother (Ⅱ2). MS-PCR showed the maternal allele was missing in the proband, while paternal allele was missing in its mother. MS-MLPA showed deletion of the *DLK1*, *MEG3*, *MIR380*, and *RTL1* genes of both the proband and its mother. *MEG3* imprinting gene methylation increased in the proband, while decreased in its mother. It was indicated that a maternally transmitted deletion was responsible for Kagami–Ogata syndrome in the proband (Ⅲ2), and the *de novo* paternal deletion resulted in Temple syndrome in the mother (Ⅱ2). Prenatal diagnosis was provided at 17^+3^ weeks of pregnancy on the mother’s fourth pregnancy (Ⅲ4). Fortunately, the karyotype and single-nucleotide polymorphism array (SNP array) results were normal. The current investigation provided the detection methods for imprinted gene diseases, expanded the phenotype spectrum of the disease, and obtained the insight into the diagnosis, prenatal diagnosis, and genetic counseling of the disease.

## Introduction

Genomic imprinting is an epigenetic marking phenomenon that allows gene expression predominantly from a single parental allele (Reik and Walter, 2001; Eggermann et al., 2015; Soellner et al., 2017). Disturbances of the human chromosome 14q32 imprinted domain are associated with Temple syndrome (TS) (OMIM 616222) and Kagami–Ogata syndrome (KOS) (OMIM 608149). The imprinted genes are either exclusively expressed from the maternal (e.g., *MEG3*, *RTL1as*, and *MEG8*) or paternal allele (e.g., *DLK1* and *RTL1*) (van der Werf et al., 2016). They harbor the germline-derived primary *DLK1*-*MEG3* intergenic differentially methylated region (IG-DMR) and the postfertilization-derived secondary *MEG3*-DMR, as well as *MEG8*-DMR, together with multiple imprinted genes (van der Werf et al., 2016; Prasasya et al., 2020). The IG-DMR regulates the methylation status of the *MEG3*-DMR (Kagami et al., 2010; Beygo et al., 2015).

Buiting et al. (2008) named TS to describe the first patient with UPD(14)mat and another with a *DLK1*/*GTL2* epimutation. KOS is caused by UPD(14)pat, epimutations, and microdeletions affecting the IG-DMR and/or the *MEG3*-DMR of maternal origin (Huang et al., 2019). Both TS and KOS are recognized congenital diseases resulting from an abnormal dosage of imprinted genes. Li et al. (2021) reported that among all published KOS cases, >60% were due to UPD(14)pat, nearly 25% were caused by microdeletions, and about 10% were derived from epimutations of the chromosome 14q32 imprinted region. Kagami et al. (2017) reported a relative frequency of 72% UPD(14)mat, 19% epimutations, and 9% microdeletions as underlying causes of TS.

Here, we described a fetal case of KOS due to the maternal deletion of 732.26 kb at 14q32.2q32.31 and its mother with Temple syndrome because of *de novo* deletion at her paternal allele.

## Patients and methods

### Case presentation

A 25-year-old Chinese woman was referred to the Department of Reproductive Genetics, Women’s Hospital, School of Medicine, Zhejiang University. At 29^+5^ weeks of gestation during her second pregnancy, she underwent an ultrasound which showed an evident polyhydramnios, with the deepest vertical pocket (DVP) at 93 mm. At 32^+3^ weeks, another ultrasound revealed gastroschisis and an amniotic fluid index (AFI) of 313 mm. The pregnancy was terminated at the 32nd week of gestation after genetic counseling, and samples were collected from the fetal tissue. The couple denied consanguinity and had no familial history of congenital anomalies. The pregnant woman had no exposure to drugs or radiation during pregnancy.

She had four pregnancies. The first fetus (Ⅲ1) was terminated by artificial abortion on her request at 7^th^ week, and the third fetus (Ⅲ3) was revealed as a biochemical pregnancy. It is worth noting that the 75 g oral glucose tolerance test indicated much higher blood glucose levels than normal pregnant women in her two pregnancies (Ⅲ2, Ⅲ4); fasting plasma glucose was 6.62 mmol/L and 6.88 mmol/L (reference range: 3.89–6.11 mmol/L), 1-h glucose was 13.94 mmol/L and 13.64 mmol/L (reference range: 7.7–8.9 mmol/L), and 2-h glucose was 11.48 mmol/L and 12.40 mmol/L (reference range: 3.89–7.8 mmol/L), respectively.

**FIGURE 1:**
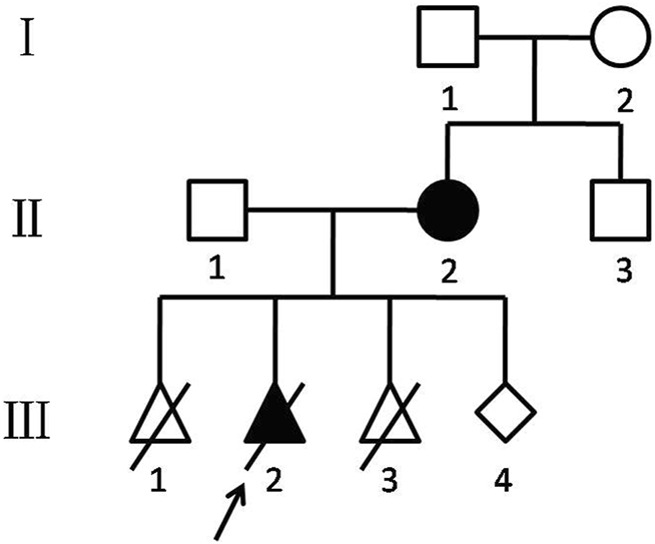
Chinese KOS/TS pedigree. Black-filled symbols indicate patients who suffered from TS (Ⅱ2) and KOS (Ⅲ2). The proband (Ⅲ2) is indicated by an arrow. Ⅲ1 was ended by artificial abortion at 7 weeks. Ⅲ2 was induced labor at 32 weeks. Ⅲ3 was revealed as a biochemical pregnancy.

All family members (Figure1) except the fetus (Ⅲ1, Ⅲ3, and Ⅲ4) were referred for copy number variation sequencing (CNV-seq) analysis to investigate the etiology of this disease.

### Copy number variation sequencing (CNV-seq)

Genomic DNA was extracted from fetal tissues (Ⅲ2) and peripheral blood (Ⅰ1, Ⅰ2, Ⅱ1, Ⅱ2, and Ⅱ3) with the Gentra Puregene Kit (QIAGEN, Hilden, Germany). CNV-seq analysis was performed using a Universal Sequencing Reaction Kit (combined probe anchored polymeric sequencing; WuHan MGI Tech Co., Ltd., China), according to the manufacturer’s instructions. The variants were annotated using databases including DGV, DECIPHER, OMIM, ClinGen, and ClinVar.

### Methylation-specific PCR (MS-PCR)

The DNA Bisulfite Conversion Kit (DP125; TIANGEN, China) was used to amplify the methylated and unmethylated *MEG3*-DMR and accurately identified normal, TS, and KOS in bisulfite-converted DNA samples. Two independent primer sets were used to amplify the unmethylated and methylated DMR, as previously described (Murphy et al., 2003). As illustrated in Table 1, UF and UR primers were used to amplify unmethylated DNA to produce a 120-bp band in normal or TS samples, while the MF&MR primers specific for methylated DNA would produce a 160-bp band in normal or KOS samples. The M and U primers and the reaction were performed on a TaKaRa Ex Taq HS enzyme (TaKaRa Bio Inc). The PCR program was as follows: 98°C for 5min, followed by 10 cycles at 98°C for 10s, 62°C for 30s, and 72°C for 30 s; then another 25 cycles at 98°C for 10s, 60°C for 30 s, and 72°C for 30 s; and a final extension at 72°C for 10 min.

**TABLE 1:** MS-PCR primers and PCR product size.

Primer	Primer sequence	PCR product size
Methylated-forward (MF)	GTT​AGT​AAT​CGG​GTT​TGT​CGG​C	160bp
Methylated-reverse (MR)	AAT​CAT​AAC​TCC​GAA​CAC​CCG​CG	
Unmethylated-forward (UF)	GAG​GAT​GGT​TAG​TTA​TTG​GGG​T	120bp
Unmethylated-reverse (UR)	CCA​CCA​TAA​CCA​ACA​CCC​TAT​AAT​CAC​A	

M and U primers, specific to bisulfite-converted methylated and unmethylated DNA, respectively.

### Methylation-specific multiplex ligation-dependent probe amplification (MS-MLPA)

The SALSA MS-MLPA kit ME032 (MRC Holland, Amsterdam, Netherlands) was used for MS-MLPA analysis. It contains 46 (MS-) MLPA probes including three for the 14q32.31 region and nine for the 14q32.2 region. Three of these probes contain a HhaI recognition site that can provide information about the methylation status of the 14q32 region. Although the probe for the *MIR380* region is a HhaI recognition site, it will only provide information on copy number changes since this HhaI recognition site is fully methylated in normal tissue. This probemix can also be used to detect *DLK1*/*MEG3*/*RLT1*/*MIR380* gene dosage in the analyzed sample. Two digestion control probes were designed to understand whether digestion in the MS-MLPA reaction was completed. The assay was performed according to the manufacturer’s instruction, and the PCR products were resolved on an ABI Prism 3730 Genetic Analyzer (Applied Biosystems, CA, United States) by Coffalyser.Net software (http://www.coffalyser.net).

### Amniocentesis and fetal karyotyping

Amniocentesis of the fetus (Ⅲ4) was performed at 17^+3^ weeks with real-time ultrasound guidance. A measure of 30 ml of amniotic fluid was collected, and the initial 5 ml was discarded. Amniotic fluid cells were cultured with BIO-AMF-2 Complete Medium (Biological Industries, Cromwell, CT) in a 5% CO_2_ incubator at 37°C. G-band analysis at 320–400 band resolution was performed on the cultured cells, according to the standard procedure.

### Single-nucleotide polymorphism array (SNP array)

Genomic DNA of the fetus (Ⅲ4) was extracted from amniotic fluid cells with the Gentra Puregene Kit (QIAGEN, Hilden, Germany). CytoScan TM HD array (Affymetrix, Santa Clara, CA) was used to analyze the gene copy number, according to the manufacturer’s instruction. Chromosome Analysis Suite software (Affymetrix, Santa Clara, CA) was used to analyze the raw data and visualize the results based on the GRCh37/hg19 assembly.

## Results

### CNV-seq results

**FIGURE 2:**
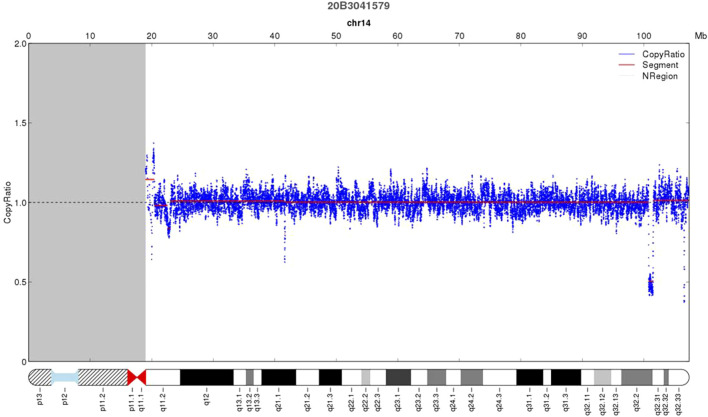
CNV-Seq result showing a 739 kb deletion of 14q32.2q32.31 in the proband (Ⅲ2).

As shown in Figure 2, deletion of 732.26 kb was detected by CNV-seq at 14q32.2q32.31 in the proband (Ⅲ2) (46,XN,del (14q32.2q32.31).seq [GRCh37/hg19](100,765,430–101,497,691)×1), which was inherited from the mother. The deleted locus contained *DLK1*, *MEG3*, *RTL1*, and *MEG8*. No deletion was observed in the woman’s parents and her younger brother, indicating that the woman carried a *de novo* deletion.

### MS-PCR results

**FIGURE 3:**
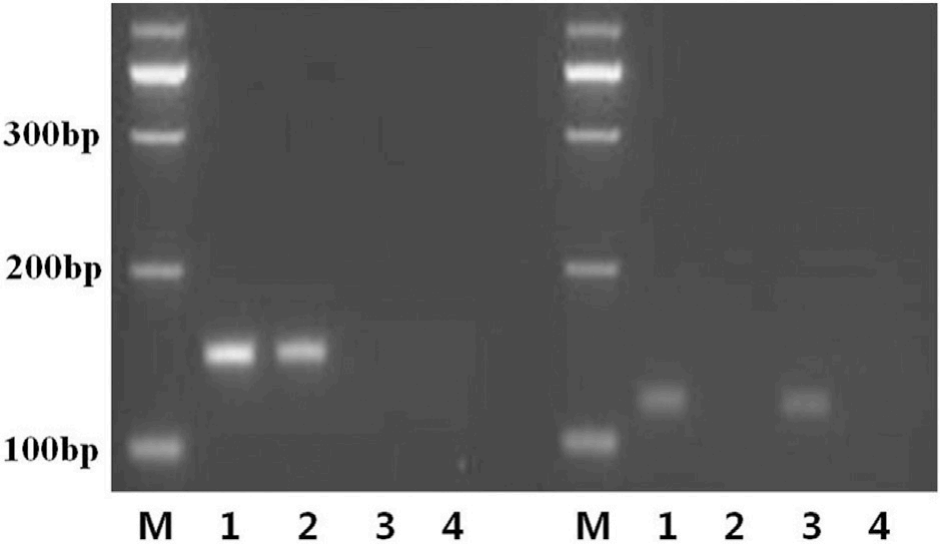
MS-PCR primers specifically designed to amplify the methylated and unmethylated copies of the *MEG3-DMR*. Bisulfite-treated or -untreated genomic DNA was subjected to MS-PCR using the M or U primer pairs separately or multiplexed to generate a 160-bp or 120-bp band from bisulfite-modified methylated (left lanes 1, 2) and unmethylated (right lanes 1 and 3) template DNA, respectively. Sample 1: normal control; sample 2: the proband; sample 3: the mother; sample 4: negative control; M: a 100-bp size ladder.

UF and UR and MF and MR primers were used to amplify samples from a normal individual, the proband (Ⅲ2), mother, and negative control. We speculated that the mother may have only unmethylated (maternal) *MEG3* that might cause TS. As shown in Figure 3, the MF and MR primers specific for methylated DNA produced a 160-bp band in the normal and proband’s samples (left lanes 1 and 2), while the UF and UR primers designed to amplify unmethylated DNA produced a 120-bp band in the normal individual and the mother’s samples (right lanes 1 and 3). The proband produced only methylated *MEG3*-DMR, while the proband’s mother produced only unmethylated *MEG3*-DMR, as anticipated, indicating loss of the paternal *MEG3*-DMR.

### MS-MLPA results

**FIGURE 4:**
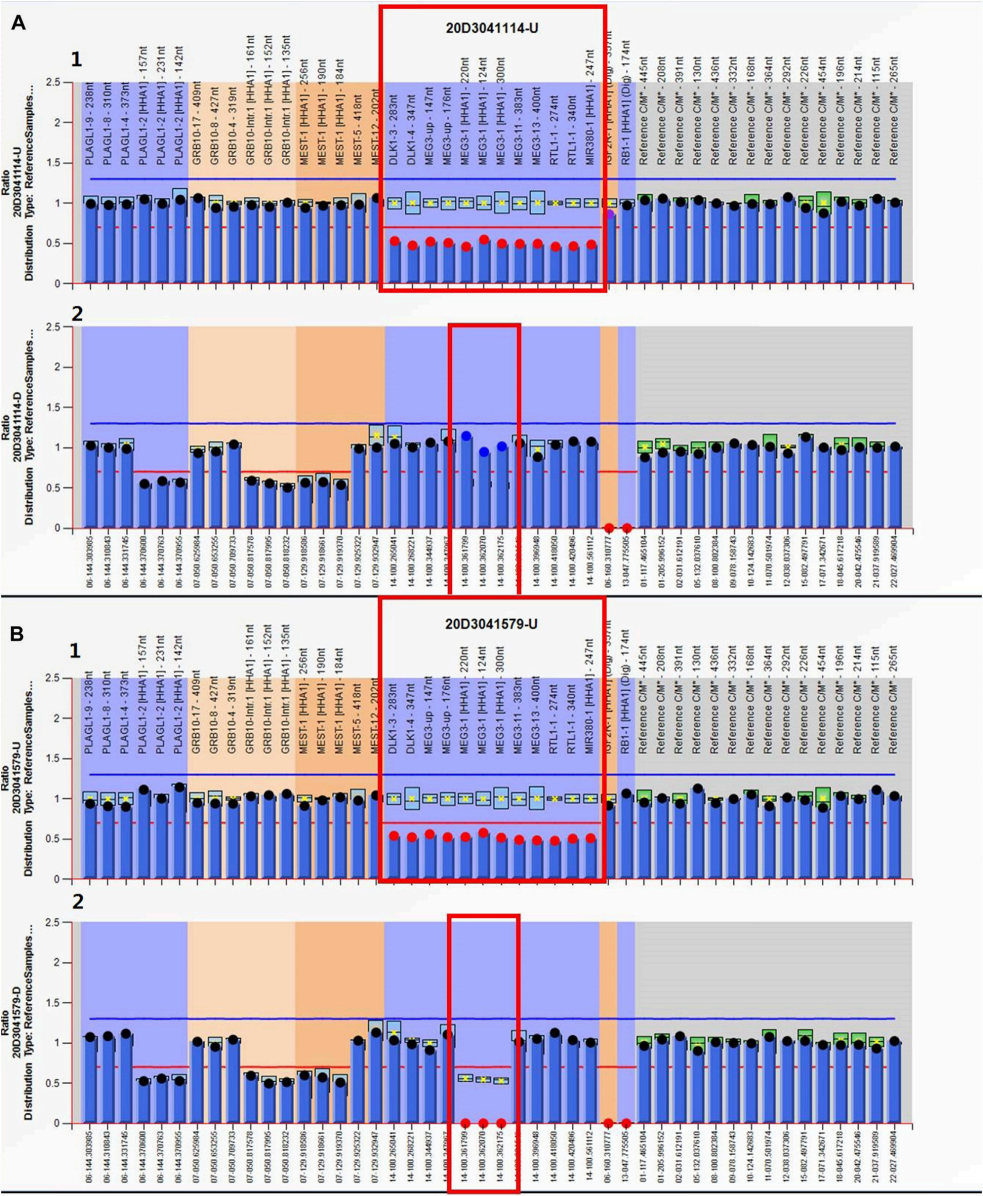
MS-MLPA results. Electropherograms and normalized data for the **(A)** proband and **(B)** the mother. (A1, B1) Copy number ratio of the 14q32 region was ∼0.5 for both cases (before HhaI digestion) (red box). Three of these probes contain a HhaI recognition site that can provide information about *MEG3*’s methylation status. (A2, B2) Methylation ratio of the *MEG3* of chromosome 14q32 after digestion was ∼1 for the proband and ∼0 for its mother (red box).

MS-MLPA analysis was used to validate the CNV-seq results. Copy number changes with a peak ratio value ∼0.5 (one copy) at the 14q32 region were observed in both the proband and its mother (Figure 4). The methylation ratios at *MEG3* of both were, respectively, ∼1 and ∼0 in comparison with the ∼0.5 methylation ratio from a normal control. This indicated that the proband suffered from a deletion of the KOS critical region, while the mother was a TS deletion patient.

### Karyotype analysis and single-nucleotide polymorphism array (SNP array) results

Fetal (Ⅲ4) karyotype analysis and SNP array did not reveal any abnormality.

## Discussion

Herein, we described a case (proband, Ⅲ2) diagnosed with KOS due to the maternal deletion of 14q32.2q32.31. The proband (Ⅲ2) showed polyhydramnios and gastroschisis, consistent with the suspicion of KOS, and was confirmed in molecular analyses. MS-PCR and MS-MLPA determined that the deletion of the paternal chromosome was sufficient to cause the TS phenotype in the proband’s mother.

Prasasya et al.’s (2020) reviewed prenatal findings included polyhydramnios, omphalocele, macrosomia, and placentomegaly and other clinical findings with KOS. In our case, the ultrasound scan revealed gastroschisis and polyhydramnios in the proband. The parents were referred for CNV-seq to search for the genetic cause and showed a 732.26 kb deletion in the proband (Ⅲ2), and we found that the microdeletion was inherited from the mother. However, the genotypes of the mother’s parents (Ⅰ1, Ⅰ2) and her younger brother (Ⅱ3) were normal. The microdeletion included one OMIM pathogenic gene (*WARS1*) and three maternally expressed genes (*MEG3*, *RTL1as*, and *MEG8*), as well as two paternally expressed genes (*DLK1* and *RTL1*). Microdeletions on 14q32, resulting in KOS and TS, have been reported. The clinical phenotype of the proband was consistent with a KOS’ intrauterine phenotype. Table 2 summarizes the prenatal ultrasound findings on previously reported KOS cases.

**TABLE 2:** Summary of prenatal ultrasound findings on previously reported cases of KOS.

References		Polyhydramnios	Omphalocele	Gastroschisis	Other phenotypes
Current study		**+**	**-**	**+**	**-**
Sargar et al. (2014)		**+**	**+**	NA	Arthrogryposis
Beygo et al. (2015)	Patient 1	**+**	NA	NA	NA
	Patient 2	**+**	NA	NA	NA
	Patient 3	**+**	NA	NA	NA
Watanabe et al. (2015)		**+**	NA	NA	Narrow thorax; MRI showed a small bell-shaped thorax
Corsello et al. (2015)		**+**	NA	NA	NA
Schmeh et al. (2016)		**+**	NA	NA	NA
Yuan et al. (2016)		**+**	NA	NA	NA
Vecchio et al. (2016)		**+**	NA	NA	Fetal bradycardia
van der Werf et al. (2016)	Patient AⅡ2	**+**	NA	Abdominal wall defects	Bell-shaped, narrow thoracic deformity, and distal arthrogryposis deformities
	Patient BⅡ1	**+**	NA	NA	NA
	Patient BⅡ3	**+**	NA	NA	Sonographically suspected encephalocele (not confirmed postnatally)
Luk (2017)	Elder sister	**+**	NA	NA	Short limbs and a small chest
	Younger brother	**+**	NA	NA	Short limbs and a narrow chest
Haug et al. (2018)		**+**	NA	NA	NA
Chen et al. (2019)		**+**	**+**	NA	Abnormal spine curvature, skin edema, and ventricular septal defect
Yamagata et al. (2018)		**+**	NA	NA	NA
Jung et al. (2018)		+	NA	NA	Small stomach bubble, mild left ventriculomegaly, and macroglossia
Huang et al. (2019)		**+**	**+**	NA	Estimated fetal weight large for date
Igreja da Silva et al. (2019)		**+**	**+**	NA	Skeletal deformities (short limbs, arcuate ulna, and indirect signs of joint contractures)
Altmann et al. (2020)		**+**	**+**	NA	Macrosomia (above the 97th percentile) and abnormal facial features (a prefrontal edema and a flat facial profile)
Al-Mudares and Fernandes. (2020)		**+**	**+**	NA	NA
Sakaria et al. (2021)	Patient 1	**+**	**+**	NA	Overlapping digits and rocker bottom feet
	Patient 2	**+**	Suspected	NA	NA

NA, data not available.

Polyhydramnios is the most common prenatal finding in patients with KOS. As shown in Table 2, all cases presented polyhydramnios. In the review by Curtis et al. (2006), all 14 cases showed this feature as well. Li et al. (2021) reviewed prenatal ultrasound findings of KOS in 33 cases, and almost all showed polyhydramnios. Although the earliest reported polyhydramnios by Chen et al. (2019) was at 18 weeks of gestation, our proband presented with polyhydramnios at 29^+5^ weeks and with gastroschisis at 32^+3^ weeks. This is different from most patients with KOS. Except for polyhydramnios, the other most common prenatal findings by ultrasound or MRI included an omphalocele (eight cases), small or narrow thorax, short limbs, and small or absent stomach. According to previous reports, Towner et al. (2001) reported the earliest ultrasound findings at 14 weeks of gestation, including an abdominal wall bulge and increased nuchal translucency (4.7 mm), possibly representing an omphalocele.

The prognosis of KOS remains poor. Prenatal diagnosis of KOS is critical and enables the parents to make informed decisions regarding both pregnancy management and postnatal care because more than 30% of KOS patients die shortly after birth or during early infancy (Ogata and Kagami, 2016). Respiratory distress is the primary cause of morbidity and mortality in patients with KOS. Mortality was reported to be 29.7% (22/74). Most deaths occurred between 2 h and 9 months. Most patients with KOS invariably have developmental delays and feeding difficulties. Other long-term complications may include seizure disorder and the need for tracheostomy and/or gastrostomy tubes (Sakaria et al., 2021).

The clinical features of TS children include low birth weight, hypotonia, motor delay, feeding problems, and facial features ranging from mild to moderate. The development phenotype is also highly heterogeneous, ranging from normal to severely delayed. Some patients show truncal obesity and skeletal findings, including small hands and/or feet, body asymmetry, kyphoscoliosis, joint hypermobility, or clinodactyly. Most affected individuals had precocious puberty and advanced bone age (Prasasya et al., 2020). Before performing genetic tests on the mother, we considered her healthy. She (Ⅱ2) was 154 cm tall, whereas her mother was 170 cm, and her father was 168 cm. Her younger brother was 165 cm. Her non-gestational weight was 52.8 kg and had a body mass index of 22.26, within the normal range. Her birth weight was 3 kg and showed normal development after birth. The age of menarche was 13 years. She graduated from domestic undergraduate school and was working as a teacher in a training institution. Afterward, we got her CNV-seq result, and after our careful inquiry, she recalled that when she was 7 years old, she went to a hospital in Shanghai for treatment because of breast development and older bone age. Considering the aforementioned clinical phenotypes, the mother (Ⅱ2) only showed precocious puberty, which was related to TS, and she did not show other phenotypes such as hypotonia, mental retardation, language retardation, and feeding problems. Her mild clinical phenotype was consistent with TS. Her parent’s CNV-seq results were normal; therefore her 14q32.2q32.31 microdeletion was *de novo*. We were unable to determine whether the 14q32.2q32.31 microdeletion was paternal or maternal. To validate the results of CNV-seq, MS-PCR and MS-MLPA were performed simultaneously. MS-PCR showed that the proband’s (Ⅲ2) amplification product was located at the 160-bp band, while the mother’s was located at the 120-bp band. Therefore, maternal PCR products were missing in the fetus, and paternal PCR products were missing in the mother. Genotypes are assigned based on the copy number ratio and their corresponding methylation ratio as determined by using the MS-MLPA kit for each sample. The copy number ratio of ∼0.5 with a methylation ratio of ∼1 (paternal allele only) or ∼0 (maternal allele only) represents either a KOS or TS deletion, respectively. The fetal copy number ratio of ∼0.5 and the methylation ratio of ∼1 suggested that the fetus was the KOS patient with a maternal allele deletion, and consequently, the mother was a TS patient with a paternal allele deletion. MS-PCR and MS-MLPA results revealed a paternal 14q32.2q32.31 microdeletion in the mother.

Based on our results, the maternally transmitted deletion was responsible for KOS in the proband. Thus, the *de novo* paternal microdeletion in the mother could have resulted in TS.

**FIGURE 5:**
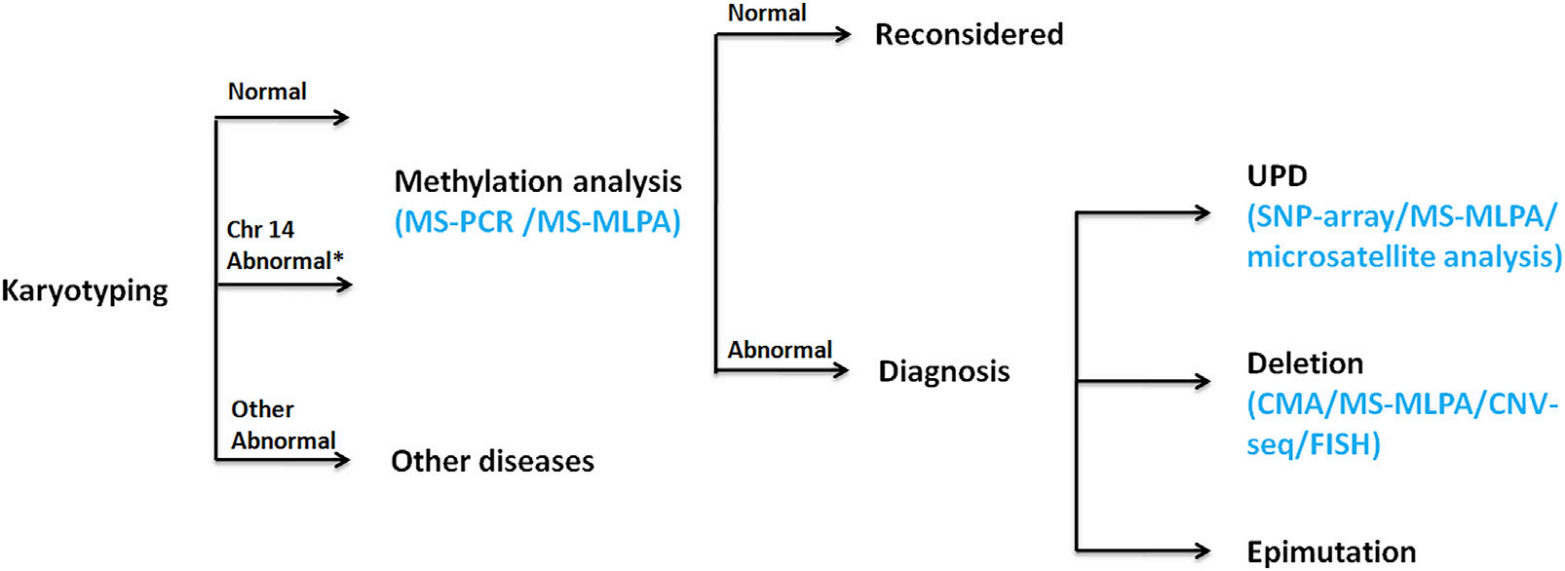
Molecular diagnostic flow chart. *Chr14 Abnormal: abnormality on chromosome 14.

A prenatal diagnosis of KOS or TS based on intrauterine phenotypes may be difficult, especially if symptoms are mild. KOS can be considered a disorder of overgrowth, with some prenatal features (e.g., placentomegaly, omphalocele, and fetal macrosomia). Molecular testing that would collectively analyze and distinguish among KOS/TS is needed. When a fetus is considered to be of KOS/TS, karyotyping should be performed as the first step, which is recommended to examine the possibility of Robertsonian translocation or other structural abnormalities on chromosome 14. Regional analysis of the DNA methylation status must be performed as the second step for each locus using MS-MLPA or MS-PCR due to almost all patients with KOS/TS having hypermethylation/hypomethylation of the IG-DMR and/or the *MEG3*-DMR. UPD can be detected by SNP array or microsatellite analysis. Deletion analysis might be performed by chromosome microarray (CMA) (including SNP array), CNV-seq, MLPA, or fluorescence *in situ* hybridization (FISH). If hypermethylation/hypomethylation is absent, clinical diagnosis should be reconsidered. The flow chart of molecular diagnosis is shown in Figure 5. As such, the use of the aforementioned tests could help prognositicate and diagnose KOS in future pregnancies as carried out in Ⅲ4; however, the utility for diagnosis and informing the parents regarding the prognosis of current pregnancy remains minimal.

In conclusion, we summarized KOS prenatal ultrasound findings to raise awareness of this condition. Our study illustrates the importance of an accurate genetic testing after ultrasound diagnosis of fetal anomalies to detect rare congenital syndromes. The value of this study lies in providing various advanced detection methods for imprinted gene diseases, expanding the clinical manifestation spectrum of the disease, and providing a good reference value for the diagnosis, prenatal diagnosis, genetic counseling, and prognosis of the disease.

## Data Availability

The datasets for this article are not publicly available due to concerns regarding participant/patient anonymity. Requests to access the datasets should be directed to the corresponding author.
